# Bariatric Surgery, Polycystic Ovary Syndrome, and Infertility

**DOI:** 10.1155/2016/1871594

**Published:** 2016-11-14

**Authors:** James Butterworth, Jean Deguara, Cynthia-Michelle Borg

**Affiliations:** ^1^University Hospital Lewisham, Lewisham and Greenwich NHS Trust, London, UK; ^2^Kingston Hospital NHS Foundation Trust, Kingston upon Thames, UK

## Abstract

*Background.* Polycystic ovary syndrome (PCOS) is the commonest cause of female infertility. Visceral obesity and insulin resistance are key pathophysiological mechanisms behind PCOS. Women suffering from this syndrome and infertility often seek bariatric surgery hoping that they would be able to conceive postoperatively.* Objective.* At present, there is no consensus on the role of bariatric surgery in the management of PCOS-associated infertility within the medical community, making it difficult to give specific advice to these women, so a review of the literature was necessary.* Results.* A detailed review of the literature was performed. Only 6 manuscripts were relevant and contained quantitative data. They demonstrated that bariatric surgery results in postoperative conception rates varying from 33% to 100%. Surgery is also associated with amelioration of menstrual irregularities, hormonal abnormalities, and hirsutism that are associated with PCOS. These studies were retrospective and only had a small number of participants with infertility.* Conclusions.* Bariatric surgery has been shown to conclusively improve life expectancy, quality of life, and comorbidities like type 2 diabetes and obstructive sleep apnea. However, further research is required to identify whether weight loss surgery results in significant improvement in fertility of women with PCOS and to investigate which operation has the best results.

## 1. Introduction

Polycystic ovary syndrome (PCOS) is the most frequent cause of female infertility. The Rotterdam criteria are often used to make a diagnosis of PCOS. These include the presence of at least two of the following: clinical and/or biochemical features of hyperandrogenism, menstrual dysfunction, and the appearance of polycystic ovaries on ultrasound, once other endocrine conditions have been excluded [1]. Other criteria that can be used include those from the National Institutes of Health (NIH) and the Androgen Excess Society (AES) [2]. PCOS has estimated prevalence of over 10% in women of childbearing age [2]. Besides being associated with infertility, PCOS is also associated with a higher incidence of type 2 diabetes mellitus (T2D), endometrial carcinoma, and cardiovascular disease including stroke and coronary heart disease.

The exact etiology of PCOS is unknown and probably represents a complex interaction between environmental and genetic factors. Insulin resistance and hyperinsulinaemia are thought to be key pathophysiological mechanisms. More than 50% of females with this syndrome are obese. Obesity in women of childbearing age is associated with anovulation, infertility, pregnancy loss, pregnancy-associated complications such as preeclampsia and gestational diabetes, and postpartum complications including hemorrhage as well as higher rates of infant mortality and congenital defects [3, 4]. Obesity in patients with PCOS is also associated with delayed or failed response to fertility treatments including clomiphene citrate, gonadotropins, and assisted insemination [5, 6]. The British Fertility Society advises that fertility treatment should be deferred until women have a body mass index (BMI) of less than 35 or BMI under 30 if they are below 37 years of age [7]. Metformin and nonsurgical weight loss measures have been advocated as first-line management for PCOS [8, 9]. It has been suggested that even a modest loss of up to 5% of the initial body weight can result in spontaneous ovulation, restoration of menstrual cycle regularity, and pregnancy in obese women with PCOS [10–12].

Bariatric surgery is the most durable and effective treatment for morbid obesity and also results in the improvement of the metabolic syndrome. With the safety of the laparoscopic approach and improved understanding of the metabolic changes occurring in bariatric patients postoperatively, morbidly obese women with infertility secondary to PCOS have resorted to bariatric surgery [13]. Historically, epidemiological studies have suggested that the rapid weight loss in the first year or two after bariatric surgery may increase women's chance of conception. While the incidence of PCOS decreases significantly after surgery [14], there are very few studies assessing fertility before and after bariatric operations. At present, there is no consensus on the role of such surgery in the management of infertility and whether surgery can also be beneficial in women who have a BMI of under 40 kg/m^2^.

In this article, we systematically review the published literature to assess the effects of bariatric surgery on fertility in women with PCOS.

## 2. Materials and Methods

### 2.1. Protocol and Registration

The PRISMA Statement for Reporting Systematic Reviews and Meta-Analyses of Studies that Evaluate Health Care Interventions: Explanation and Elaboration was utilized as a framework for this systematic review [15].

### 2.2. Eligibility Criteria

All manuscripts assessing the quantitative effect of gastric bypass, gastric banding, sleeve gastrectomy, and gastric plication on infertility in females with PCOS published between 1 January 1974 and 20 March 2015 were considered eligible for inclusion in this systematic review. Studies involving vertical banded gastroplasty and biliopancreatic diversion were not included.

### 2.3. Information Sources and Search

Search databases, PubMed, Embase from 1974 to 20 March 2015, and MEDLINE and MEDLINE Non-Indexed Items, were searched using the following keywords: polycystic ovary syndrome, infertility, bariatric surgery, gastric bypass, laparoscopic, Roux-en-Y, gastric band, sleeve gastrectomy, and gastric plication. Reference lists were also scanned for relevant manuscripts.

### 2.4. Study Selection

Studies identified were screened for relevance and suitability by the two authors. Manuscripts lacking quantitative data, those lacking in relevance to the study question, and those relating to male fertility were excluded.

### 2.5. Data Collection Process

Results and data were extracted following analysis and critical review of the results section of original manuscripts.

### 2.6. Data Items

*Participant Information.* This included sample numbers, age, body mass index, and basic demographics.

*Surgical Procedure and Technique.* This list included Roux-en-Y gastric bypass (RYGB), gastric band (GB), gastric plication (GP), or sleeve gastrectomy (SG), open or laparoscopic.

*Comparisons.* Comparisons were done for epidemiological studies identified.

*Outcomes.* Outcomes were conception rate, pregnancy, biochemical markers of fertility and PCOS, and menstrual regularity.

*Study Design.* The type of study and level of evidence were recorded.

### 2.7. Risk of Bias in Individual Studies

Each study was individually assessed for risk of bias giving particular attention to funding sources, limitations of study, and conflicts of interest declared in the discussion section.

### 2.8. Summary Measures

As the literature primarily is composed of epidemiological studies, the principle summary measure is sample conception rates before and following surgery in addition to/or other biochemical markers of fertility and PCOS within the same sample.

### 2.9. Synthesis of Results

Results of studies have been summarized; however, quantitative data have not been combined in analysis.

## 3. Results and Discussion 

68 manuscripts were identified which met the search criteria. Out of these, 6 were included in analysis as they were relevant and contained quantitative data. 19 manuscripts, despite being relevant, had no quantitative data. 43 manuscripts were not relevant. The 6 manuscripts that were included in the analysis and their results are summarized in Table 1.

Eid et al. [16] demonstrated that, after laparoscopic RYGB (75 cm Roux limb), weight loss was associated with amelioration of PCOS-associated symptoms including resolution of menstrual abnormalities in all patients and resolution of hirsutism in 52% of patients. Surgery also resulted in resolution of T2D and improvement in hypertension and dyslipidaemia. Five patients who were unable to conceive preoperatively were able to conceive without the use of hormones postoperatively although the time interval after surgery is not mentioned in this paper.

In a study by Jamal et al. [17], 20 patients with PCOS were followed up after RYGB (75 cm Roux limb and 30 cm biliopancreatic limb) for a mean postoperative follow-up of 46.7 months. Preoperatively, 50% of the patients with PCOS were infertile, 85% had menstrual dysfunction, and 70% had hirsutism. Following surgery-induced weight loss, menstrual irregularities were corrected with return of regular cycles in 82% of patients without the need for hormonal treatment. Hirsutism completely resolved in 29% and 77.8% of those with T2D that had complete remission. Of the 10 patients who did not conceive before surgery, 4 no longer desired pregnancy with the remaining 6 patients becoming pregnant within 3 years of surgery (5 of whom conceived without any hormonal treatment). These patients did not develop any pregnancy induced or postpartum complications.

In a case series of 5 patients who underwent IVF after bariatric surgery, two women with infertility secondary to PCOS and male infertility underwent RYGB and gastric banding, respectively [18]. Both conceived postoperatively following in vitro fertilization/intracytoplasmic sperm insemination and had uncomplicated deliveries. This paper suggests that although IVF appears to be safe after bariatric surgery, ovarian hyperstimulation may present with features similar to complications after weight loss surgery (especially internal herniation after RYGB) and a high index of suspicion is thus required.

Stroh et al. [13] described the progress of 3 patients who underwent gastric banding. One of these patients conceived postoperatively. Talebpour reported that gastric plication and gastric bypass appear to have a positive effect on fertility in an abstract presented at IFSO 2011 [18]. 10 women (71%) out of 14 who were infertile preoperatively became pregnant one year postoperatively. Out of 87 premenopausal patients who had irregular menstrual cycles preoperatively, 70 (80%) had regular cycles by the end of the first year postoperatively [18]. This work, only published in abstract form, does not compare the effects of the two operations.

In another abstract presented by George and Azeez at IFSO 2013 [20], 156 female patients between the ages of 20 and 50 underwent laparoscopic sleeve gastrectomy (SG) and were followed up 6-monthly. Postoperatively, hirsutism resolved in 77 out of 96 patients (80%) and 54 out of 67 showed resolution of radiological evidence of PCOS. 132 patients had menstrual irregularities before surgery but all of these returned to a normal menstrual pattern after SG. Four out of the 11 patients who were unsuccessfully treated for infertility preoperatively became pregnant postoperatively. This abstract also showed that urinary stress incontinence resolved or substantially improved in over 40% of patients. Unfortunately, this work was only published as an abstract and there is no information regarding the mean follow-up period, the preoperative BMIs, and the nature of the study.

Dixon and O'Brien [21] found that 2 infertile patients in their cohort became pregnant after laparoscopic gastric banding. Unfortunately, the paper does not make the denominator clear and it is not possible to ascertain how many of the 28 women with primary or secondary infertility were followed up 1 year postoperatively. This paper was therefore not included in the analysis table.

All these studies were very heterogeneous and had small numbers of patients. It was thereby decided that any statistical comparison of these studies would be futile. The definition of infertility, the age of the patients, and the operations were different. These could have important effects on postoperative fertility and conception. Controls were not present in the considered papers with patients being control of themselves before and after bariatric surgery. It was difficult to carry out analysis for bias in studies that were only presented as conference abstracts. Another bias deals with one of the analyzed studies that considered in vitro fertilization (often performed for the male factor), while the others considered spontaneous pregnancy.

Women of childbearing age form a significant percentage of patients being referred for and undergoing bariatric surgery. A review of admission data from more than 1,000 US hospitals between 1998 and 2005 revealed that almost half of all patients undergoing inpatient surgical weight loss procedures were women between the ages of 18 and 45 [22]. PCOS is very common in this patient group. A recent meta-analysis has shown that PCOS decreases significantly after bariatric surgery from 45.6% preoperatively to 6.8% at 1 year postoperatively [14].

The reproductive health of female participants was investigated as part of the Longitudinal Assessment of Bariatric Surgery (LABS-2) study with a self-administered survey within 30 days preoperatively [23]. 1,538 females were included and 13% of them had been diagnosed with PCOS preoperatively. 42% of women who tried to become pregnant preoperatively in this cohort of patients experienced infertility (defined as 12 months of regular intercourse with a man without contraception but no resulting pregnancy). 65% of these patients however had at least 1 pregnancy after experiencing a period of infertility. A high rate of stillbirths was self-reported by these women with the rate being twice that expected in the USA (13.2 versus 6.2 per 1000 live births). Future pregnancies (in the postoperative period) were an important consideration to 30% of patients who were aged 18–44 and who did not report natural/surgical menopause, hysterectomy, endometrial ablation, or sterilization (personal or partner). This study revealed that women who were obese by the age of 18 were more likely to report PCOS (14.4% versus 5%) and infertility (56% versus 25%) and less likely to have ever been pregnant (75% versus 92%), compared with women whose obesity started after the age of 30. Obesity at a young age may be considered an indication for bariatric surgery in effort to prevent infertility developing in later life. The LABS-2 study is yet to report the postoperative reproductive health results in this cohort of women.

PCOS in obese patients primarily manifests itself with irregular or infrequent menstrual bleeding/amenorrhea, hirsutism, and infertility. It is thought that a 5% weight loss can result in resolution of obesity-related anovulation; however, there is little evidence that this is sufficient in morbidly obese patients [24]. In a retrospective survey, 50% of women (98 patients) aged 40 years or younger with intact uterus and ovaries had anovulatory cycles, defined as cycles of >35 days longer, prior to bariatric surgery [22]. Of these 98 patients, 70 patients (71%) had a return of normal menstrual cycles after surgery. Patients who regained ovulation postoperatively had statistically significant greater weight loss compared to those who remained anovulatory. Patients who had normal cycles preoperatively still had normal menstrual cycles postoperatively despite the weight loss.

In a prospective study of 14 females with PCOS, amelioration in clinical symptoms was associated with significant improvements in testosterone, fasting glucose, cholesterol, insulin, and triglyceride levels at 6 and 12 months after RYGB when compared to baseline [25]. Improvements in biomarkers, hirsutism, and regularity of the menstrual cycles did not correlate with the degree of weight change in this study. Escobar-Morreale et al. also showed similar improvements in total and free testosterone and amelioration of insulin resistance estimated in a prospective study of 12 premenopausal women with PCOS who underwent either RYGB or biliopancreatic diversion [26].

Traditionally, bariatric surgery has been reserved for patients with a body mass index (BMI) >40 kg/m^2^ or with BMI >35 kg/m^2^ and one or more significant comorbid conditions, when nonsurgical methods of weight loss have failed. Recently, the National Institute for Health and Care Excellence (NICE) in the UK suggested lowering the BMI down to 30 kg/m^2^ in patients with a recent diagnosis of T2D [27]. Infertility due to anovulation and PCOS amongst morbidly obese women could potentially be viewed as an additional indication for bariatric surgery. Although most studies show amelioration of PCOS postoperatively, to date, the number of studies showing improved fertility is small and they mainly consist of retrospective analysis of small cohorts of patients. As females of childbearing age make up a large percentage of patients undergoing surgery, more research is required in this area of metabolic and bariatric surgery to enable clinicians to advise these women regarding their reproductive health and fertility after surgery. Careful follow-up of these patients is required, as pregnancy is usually not advised in the first 12–18 months postoperatively.

## 4. Conclusion

Bariatric surgery results in improvement of menstrual irregularities and hirsutism and amelioration of the metabolic profile. Observational studies suggest that female fertility improves following bariatric procedures and weight loss. However, at this stage, it is difficult to recommend the lowering of BMI criteria for patients with primary infertility and PCOS and larger studies are required to confirm the effects of bariatric surgery on fertility and to determine whether different bariatric operations have different results compared to nonsurgical methods of weight loss.

## Conclusions

- Polycystic ovary syndrome (PCOS) is the most frequent cause of female infertility.
- PCOS, hirsutism, and menstrual irregularities improve after bariatric surgery.
- The evidence for improvement in fertility after bariatric surgery is still limited.
- More studies are required to understand which operation (if any) would be best for this cohort of young infertile women.

## Figures and Tables

**Table 1:** Comparison of existing studies about bariatric surgery and female fertility.

	Eid et al. SOARD 2005 [16]	Stroh et al. Zentralbl Chir 2008 (paper in German, abstract in English) [13]	Doblado et al. Ferti Steril 2010 [19]	Jamal et al. SOARD 2012 [17]	Talebpour Obes Surg 2011 (abstract only) [18]	George and Azeez Obes Surg 2013 (abstract only) [20]
Participants	24 women with PCOS	3 females with PCOS	2 females with infertility secondary to PCOS (and to male factor)	10 women with PCOS and infertility	69 premenopausal married females	156 women (67 had radiological features of PCOS and 11 were infertile)
Interventions	Laparoscopic RYGB	Laparoscopic LAGB	RYGB and gastric band, respectively	RYGB	Gastric plication or RYGB	Laparoscopic SG
Outcomes measured	Mean excess weight loss, presence of hirsutism, regular menstruation, conception rate after surgery, obesity associated comorbidities	% EWL, glucose levels, conception rate	Conception and pregnancy	Pre- and postsurgery conception rate, weight loss, hirsutism, menstrual dysfunction, obesity associated comorbidities	Regularity of menstruation and conception rate	Hirsutism, stress urinary incontinence, menstrual dysfunction, infertility
Study design	Retrospective	Not stated	Retrospective	Retrospective	Not stated	Retrospective
Mean follow-up (months)	27.5 ± 16	104	Not stated	46.7 ± 35.3	12	Not stated
Results, fertility	5 women (previously infertile) who wanted to conceive were able to do so after surgery without the use of clomiphene	One patient (33%) conceived postoperatively	Following IVF/ICSI, both women became pregnant and had uncomplicated deliveries	100% postoperative conception rate in infertile patients with PCOS who desired pregnancy	10 women (71%) out of 14 who were infertile preoperatively became pregnant after one year postoperatively	4 patients (36%) conceived without any formal fertility treatment
Results – other	Resolution of T2D, decreased number of patients with hypertension and hyperlipidemia, improvement of PCOS-associated symptoms	The 3 patients had a % EWL of 49, 67, and 41%, respectively, with glucose levels normalized postoperatively		Improvement of glycemic control, PCOS-associated symptoms, hypertension, depression, GERD, and urinary incontinence	Out of 30 patients with irregular cycles preoperatively, 14 (47%) had regular cycles by the end of 1 year postoperatively	Hirsutism and radiological evidence of PCOS resolved in 80%; menstrual dysfunction improved in 100%; urinary incontinence resolved or improved in 42%
